# Management of Benign Findings Following Local Anesthetic Transperineal Prostate Biopsy: A Single-Center Review and Comparison With Current Guidelines

**DOI:** 10.7759/cureus.92763

**Published:** 2025-09-19

**Authors:** Meyada Ali, Molu Thomas, Joanna Morton, Maryum Shafiq, Segun Adeniyi, Muhammad Sanan, Paul J Lim, Anthony Dyal, William Gallagher, Vincent Koo

**Affiliations:** 1 Urology, Worcestershire Acute Hospitals NHS Trust, Redditch, GBR

**Keywords:** atypical small acinar proliferation, benign prostate biopsy, digital rectal examination, high-grade prostatic intraepithelial neoplasia, local anesthetic transperineal biopsy, pirads score, prostate cancer diagnosis

## Abstract

Introduction

Although local anesthetic transperineal (LATP) prostate biopsy is now widely adopted, evidence remains limited, and no clear consensus exists on the optimal follow-up strategy for men with benign histology. This gap is particularly important given the increasing preference for LATP over the transrectal approach and the substantial proportion of patients who receive benign biopsy results. This study evaluated the follow-up of benign LATP biopsy findings and compared them with current international guidelines.

Methods

A retrospective analysis was conducted at Worcestershire Acute Hospitals NHS Trust on patients who underwent LATP biopsy between November 2023 and April 2024. Patients with benign histology were identified and categorized by follow-up strategy: monitored, discharged to primary care, underwent repeat biopsy, or unclear. Outcomes of repeat biopsies and associated clinical parameters were assessed.

Results

Of 616 patients, 206 (33.4%) had benign histology. Sixteen patients underwent repeat biopsy due to clinical or radiological suspicion. Among those re-biopsied, six (37.5%) were diagnosed with prostate cancer, including 2 (12.5%) with clinically significant disease (Gleason Grade Group ≥2). Immediate repeat biopsy detected cancer in 50% of cases, particularly in patients with inadequate initial sampling or Prostate Imaging-Reporting and Data System (PI-RADS) 5 lesions. Complication rates were low across the cohort, although three patients developed urosepsis.

Conclusions

LATP biopsy is safe and effective, but benign findings warrant structured follow-up due to a measurable false-negative rate. Our experience supports guideline recommendations that emphasize multidisciplinary evaluation, prostate-specific antigen kinetics, and imaging. Vigilant monitoring reduces the risk of missed cancers while avoiding unnecessary procedures.

## Introduction

Prostate cancer remains one of the most commonly diagnosed malignancies among men worldwide, and accurate diagnosis depends on histological confirmation through prostate biopsy. For decades, the transrectal ultrasound-guided (TRUS) biopsy was the dominant technique, offering convenience but carrying significant drawbacks, particularly the risk of infection, including sepsis, and suboptimal sampling of anterior prostate regions [1]. These limitations became increasingly evident in landmark studies such as the Prostate MRI Imaging Study (PROMIS), which demonstrated that TRUS biopsies could miss nearly half of clinically significant cancers [2].

Over the past decade, clinical practice has shifted toward the transperineal route, which enables more comprehensive sampling, particularly of anterior zones, while markedly reducing infection rates [1]. This evolution has been driven by multiple factors: heightened emphasis on infection prevention (especially amid antimicrobial stewardship initiatives), the integration of multiparametric MRI (mpMRI) into diagnostic pathways, and updated guidance from major organizations. Guidelines from the UK National Institute for Health and Care Excellence (NICE), the European Association of Urology (EAU), and the American Urological Association (AUA) now recommend mpMRI before biopsy and strongly support the transperineal approach, with LATP increasingly recognized as the standard of care [3-5].

Globally, adoption of local anesthetic transperineal (LATP) prostate biopsy is accelerating, supported by evidence from multicenter observational studies and, more recently, the TRANSLATE randomized trial. This trial demonstrated superior detection of clinically significant cancers with LATP compared to TRUS: LATP identified Gleason Grade Group 2 (GG2) or higher disease in 60% of patients compared with 54% using TRUS (OR 1.32; p = 0.031), confirming the advantage of LATP in biopsy-naïve men [1].

While malignant biopsy findings clearly guide treatment decisions, benign results present a clinical challenge. A benign outcome may reflect a true absence of malignancy or represent a false negative due to sampling error or tumor heterogeneity. Reported false-negative rates for LATP biopsy range from 5% to 15%, influenced by the number and distribution of biopsy cores, lesion characteristics, and operator experience [6]. Accurate interpretation of benign results requires integration with the broader clinical picture, including prostate-specific antigen (PSA) levels, PSA kinetics, digital rectal examination (DRE) findings, and mpMRI results. Persistent clinical or radiological suspicion despite a benign biopsy may warrant repeat imaging or biopsy.

Current national and international guidelines support this cautious, structured approach. The UK NICE guideline NG131 recommends pre-biopsy mpMRI and endorses the transperineal route. After a benign biopsy, NICE advises ongoing PSA monitoring, repeat MRI if PSA remains elevated, and consideration of repeat biopsy if suspicion persists [3]. Similarly, the EAU recommends mpMRI before biopsy and repeat biopsy when suspicion remains, particularly for Prostate Imaging-Reporting and Data System (PI-RADS) 4 or 5 lesions [4]. The EAU also highlights the lower infection risk and improved anterior sampling with LATP, supporting its use for both initial and repeat biopsies. The AUA endorses risk-stratified follow-up after a benign biopsy, incorporating PSA kinetics, MRI findings, and family history [5]. Importantly, the AUA stresses that a benign biopsy should not be considered definitive in the presence of rising PSA or concerning MRI findings.

All major guidelines now recommend mpMRI before biopsy and increasingly favor the transperineal approach, including LATP, for both initial and repeat procedures. Following a benign biopsy, each guideline supports a risk-adapted follow-up strategy that integrates PSA monitoring, MRI reassessment, and histological context. Triggers for repeat biopsy are broadly consistent across NICE, EAU, and AUA guidance, including persistently elevated or rising PSA, suspicious MRI findings (particularly PI-RADS 4-5), and atypical histological findings such as atypical small acinar proliferation (ASAP) or high-grade prostatic intraepithelial neoplasia (HGPIN). Our center’s protocol mirrors these recommendations, ensuring that patients with benign biopsy results are appropriately monitored while avoiding unnecessary interventions.

This study presents our single-center experience in managing benign histology following LATP prostate biopsy. The primary objective was to quantify the cancer detection rate on repeat biopsy after an initial benign LATP result. Secondary objectives were to quantify the yield of clinically significant cancer (Grade Group 2 or higher), describe complications, and assess concordance between actual management pathways and triggers recommended by contemporary guidelines. For this study, “benign” was defined strictly as histologically benign tissue, while patients with ASAP or HGPIN were analyzed separately as atypical subgroups.

## Materials and methods

Study design and setting

We conducted a retrospective review of patients who underwent LATP prostate biopsy at Worcestershire Acute Hospitals NHS Trust between November 2023 and April 2024. The study was carried out in accordance with institutional governance policies and designed as a service evaluation. The primary objective was to quantify the cancer detection rate on repeat biopsy following an initial benign LATP result. Secondary objectives were to determine the yield of clinically significant cancer (Grade Group 2 or higher), describe complications, and evaluate concordance between real-world management and triggers recommended by contemporary guidelines.

Participant selection

All men referred for prostate biopsy because of raised PSA, abnormal DRE, strong family history, or concerning mpMRI findings (PI-RADS ≥3) were eligible for LATP biopsy. There were no formal exclusion criteria; however, patients with missing follow-up data or those who transferred care outside the Trust were classified as having “unclear follow-up” to minimize selection bias. All included patients had biopsy specimens reported by consultant uropathologists and underwent follow-up in the urology department.

Biopsy procedure

All biopsies were performed using the PrecisionPoint™ freehand Transperineal Access System (Perineologic, Inc., Cumberland, MA, USA). Antibiotic prophylaxis was tailored according to local microbiology guidance and reserved for higher-risk groups, such as immunocompromised patients, those with a post-void residual >150 mL, or a history of prior biopsy. Consequently, 12% of patients received pre-procedure prophylaxis (a single dose of ciprofloxacin and gentamicin), while 13% received a short post-procedural course (a two-day ciprofloxacin regimen).

Periprostatic nerve blocks were administered using 10-20 mL of 1% lignocaine infiltrated bilaterally at the neurovascular bundles under ultrasound guidance, supplemented with 10 mL of 1% lignocaine with adrenaline for superficial skin infiltration to improve tolerance. mpMRI findings guided targeted sampling of suspicious lesions. All patients underwent systematic biopsies, with additional targeted cores obtained from any PI-RADS ≥3 lesions. On average, 23 cores were taken per patient (range 2-42), balancing adequate coverage with minimizing procedural morbidity.

Histopathological analysis

All biopsy specimens were reviewed by specialist uropathologists and reported according to the International Society of Urological Pathology (ISUP) Grade Group system for malignant findings [7]. This system categorizes tumors into five Grade Groups (1-5) based on the Gleason scoring system: Grade Group 1 corresponds to Gleason score 3+3 (low-risk disease), Grade Groups 2-3 represent intermediate-risk disease, and Grade Groups 4-5 denote high-risk cancers with increasing proportions of Gleason patterns 4 or 5.

For analytical purposes, benign histology was broadly defined as nonmalignant findings, including normal prostate tissue with or without inflammation, prostatitis, ASAP, and HGPIN. Within the benign cohort, ASAP and HGPIN were recorded as subdivisions for descriptive reporting but were not classified as malignant. This approach reflects their noncancer status while recognizing their established role as risk markers for subsequent prostate cancer.

Data collection and follow-up classification

Clinical parameters, including PSA levels, PI-RADS scores, DRE findings, and management strategies, were extracted from electronic medical records. Biopsy-specific variables included the number of cores taken, histological diagnosis (such as benign findings, ASAP, HGPIN, or prostatitis), and the ISUP Grade Group in cases where cancer was detected. Patients with benign biopsies were identified, and management outcomes were categorized as discharge, monitoring, immediate repeat biopsy, or delayed repeat biopsy. Discharge was defined as benign histology with stable PSA, nonsuspicious MRI findings, and no concerning clinical features, with patients returned to primary care. Monitoring was defined as benign histology but with persisting suspicion (elevated or rising PSA, family history, or PI-RADS 3 lesions), with PSA checks every six to 12 months and repeat MRI if warranted. An immediate repeat biopsy was undertaken within three months in cases with high suspicion of missed cancer (e.g., a PI-RADS 5 lesion not adequately sampled or MRI-histology discordance). Delayed repeat biopsy was defined as re-biopsy at ≥12 months, typically triggered by PSA rise, upgraded MRI findings, or new clinical concerns. For patients undergoing repeat biopsy, outcomes were further classified as benign or malignant, and cancers were stratified by clinical significance based on ISUP Grade Group. The presence of adverse events, including infection, urosepsis, and hospital readmission, was also recorded.

mpMRI scans were interpreted according to PI-RADS version 2.1 and reported by fellowship-trained uroradiologists. Targeted biopsies were performed using a cognitive fusion approach alongside systematic sampling. Histopathological diagnoses were reviewed by specialist uropathologists. For analytical purposes, benign histology was defined as entirely benign tissue, while ASAP and HGPIN were classified as atypical subgroups and analyzed separately from the benign cohort. All cases were discussed within a multidisciplinary team comprising urologists, uroradiologists, and uropathologists, ensuring standardized interpretation of imaging and pathology and consensus-based management decisions.

Data sources and measurement

Data for this study were retrospectively extracted from electronic medical records, including Sunrise and the ICE pathology system at Worcestershire Acute Hospitals NHS Trust. Biopsy histology reports were reviewed through the hospital’s pathology information system and interpreted by specialist uropathologists in accordance with ISUP 2014 grading criteria. PI-RADS scores were obtained from mpMRI reports issued by consultant radiologists with expertise in prostate imaging. PSA levels and other laboratory data were retrieved from the hospital’s central laboratory database. All clinical data were collected and verified by members of the clinical research team, and all datasets were anonymized before analysis.

Statistical analysis

Descriptive statistical methods were used to summarize the data. Categorical variables, such as follow-up strategy and repeat biopsy outcomes, were reported as frequencies and percentages, with exact binomial 95% CIs (Clopper-Pearson method) calculated for key proportions. Continuous variables, including age and PSA levels, were summarized using means, SDs, and ranges. No inferential statistical testing was performed due to the observational and descriptive nature of the study. Data analysis was conducted using Microsoft Excel (Microsoft Corporation, Redmond, WA, USA).

Ethical approval

This retrospective study was conducted at Worcestershire Acute Hospitals NHS Trust and was registered with the institutional clinical audit and governance department as a service evaluation (audit ID: 11782). The study involved analysis of routinely collected, anonymized clinical data and did not involve any intervention or deviation from standard care.

## Results

Between November 2023 and April 2024, 616 patients underwent LATP prostate biopsy at Worcestershire Acute Hospitals NHS Trust. LATP has been fully adopted at our center as the standard diagnostic approach, with combined targeted and systematic sampling routinely performed (average of 23 cores per procedure; range, 2-42).

**Figure 1:**
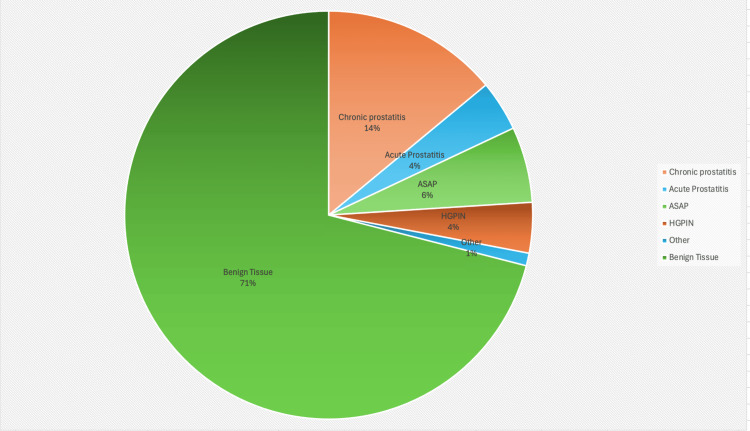
Distribution of benign histology findings Pie chart showing the breakdown of benign histology in 206 patients following LATP prostate biopsy. Most cases were benign tissue (71%), with smaller proportions of chronic prostatitis (14%), acute prostatitis (4%), ASAP (6%), HGPIN (4%), and other atypical findings (1%). ASAP, atypical small acinar proliferation; HGPIN, high-grade prostatic intraepithelial neoplasia; LATP, local anesthetic transperineal

Of the 616 biopsies, 206 (33.4%; 95% CI, 29.7-37.2) yielded benign histology. Within this benign cohort, chronic prostatitis was identified in 28 cases (13.6%; 95% CI, 9.3-19.1), acute prostatitis in nine cases (4.4%; 95% CI, 2.0-8.2), ASAP in 13 cases (6.3%; 95% CI, 3.4-10.6), and HGPIN in eight cases (3.9%; 95% CI, 1.7-7.5) (Figure 1). One case (0.5%; 95% CI, 0.0-2.7) demonstrated a small focus of adenocarcinoma that was insufficient for a definitive cancer diagnosis and was therefore classified as an atypical benign finding.

The mean PSA level among the benign cohort was 6.28 ng/mL (SD, 4.03; range, 0.16-30.0 ng/mL). In contrast, PSA values among patients ultimately diagnosed with prostate cancer ranged up to 5000 ng/mL, with a mean of 35.10 ng/mL (SD, 269.52), reflecting extreme outliers.

**Figure 2:**
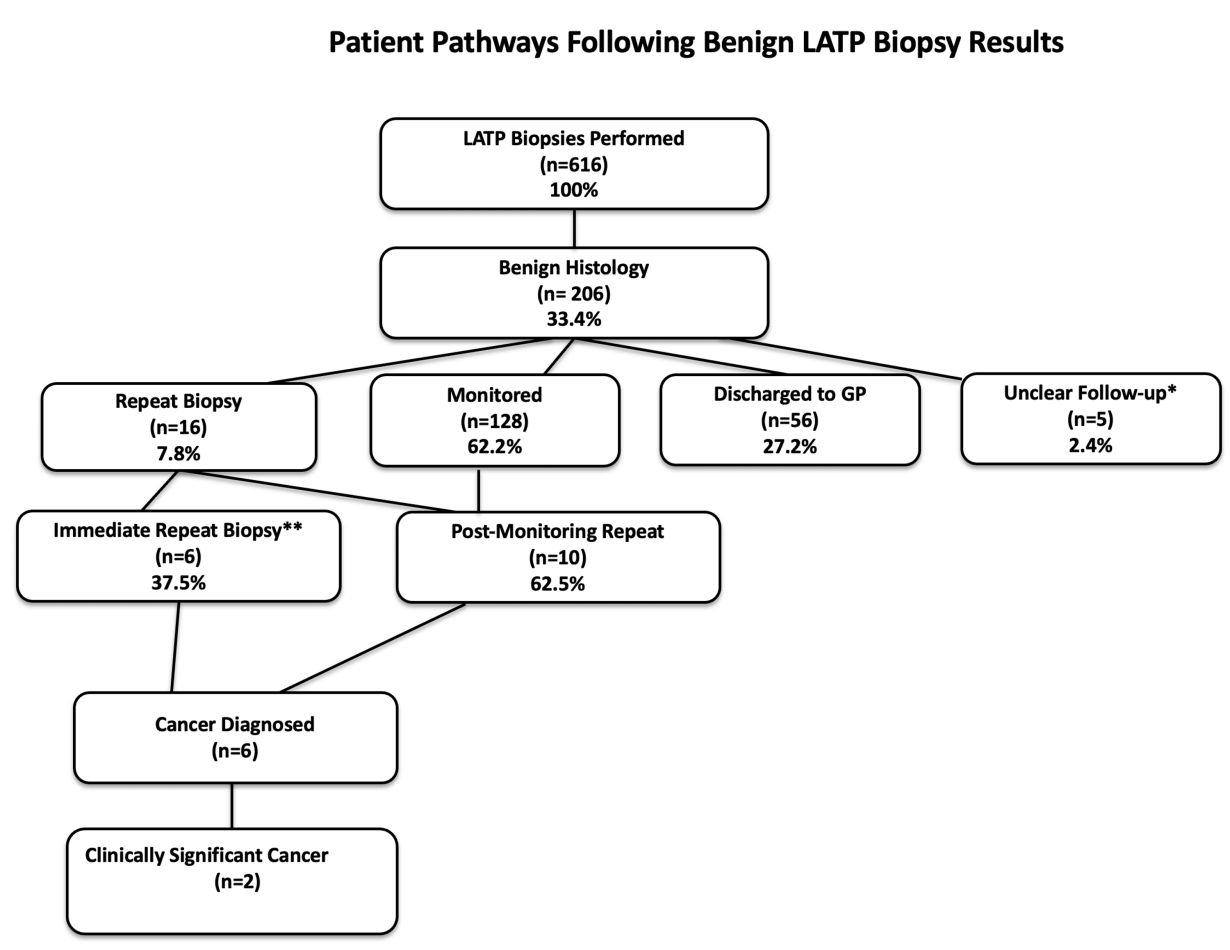
Patient pathway following benign LATP biopsy results Flowchart summarizing follow-up of 206 patients with benign LATP biopsy. Outcomes included PSA monitoring, discharged to GP, repeat biopsy, or unclear follow-up. Among the 16 patients who underwent repeat biopsy, cancer was detected in six cases, of which two represented clinically significant disease. ^*^ Unclear: Patients who self-discharged or were referred to another hospital. ^**^ Immediate repeat biopsy: Three due to inadequate initial specimens (skeletal muscle or absence of target tissue), two due to PI-RADS 5 with strong family history, and one due to small suspicious PI-RADS 4 foci. GP, general practitioner; LATP, local anesthetic transperineal; PI-RADS, Prostate Imaging-Reporting and Data System; PSA, prostate-specific antigen

Patients with benign histology were stratified into four follow-up categories (Figure 2). The majority, 128 patients (62.1%; 95% CI, 55.3-68.7), were monitored with ongoing PSA surveillance every three to six months, with mpMRI repeated if PSA levels remained elevated or rose significantly. A further 56 patients (27.2%; 95% CI, 21.2-33.9) were discharged to their general practitioner for community-based PSA monitoring, having been deemed low risk. Sixteen patients (7.8%; 95% CI, 4.5-12.3) underwent repeat biopsy, while follow-up status was unclear in five patients (2.4%; 95% CI, 0.8-5.5) who were either transferred to other centers, self-discharged, or lost to follow-up.

Overall, outcomes showed that the vast majority of benign biopsies remained benign on follow-up, while fewer than 1% were ultimately diagnosed with clinically significant prostate cancer on repeat biopsy (Table 1). 

**Table 1:** Outcomes of repeat biopsy Summary of histological outcomes in 16 patients who underwent repeat biopsy following an initial benign LATP result. Most remained benign (62.5%), while 37.5% were diagnosed with prostate cancer, including 12.5% with clinically significant disease (ISUP Grade Group ≥2). GG2, Gleason Grade Group 2; ISUP, International Society of Urological Pathology; LATP, local anesthetic transperineal

Outcome	Number of patients	Percentage of repeat biopsies
Benign on repeat biopsy	10	62.50%
Cancer	6	37.50%
Clinically significant cancer (GG2+)	2	12.50%

Monitoring subgroup

Of the 128 monitored patients (62.1% of the benign cohort; 20.8% of the total 616), 41 (32.0%; 95% CI 24.1-40.8; 6.7% of total) were eventually discharged after stable PSA and reassuring MRI findings. The remaining 87 (68.0%; 95% CI 59.2-75.9; 14.1% of total) continue under surveillance at >12 months of follow-up. Early PSA trends indicated that those ultimately discharged demonstrated stable or declining PSA, whereas those still under surveillance often had fluctuating or rising PSA, prompting ongoing monitoring.

PSA distribution and repeat-biopsy outcomes

Among the 16 patients who underwent a repeat biopsy (7.8% of the benign cohort; 2.6% of the total), six (37.5%; 95% CI 15.2-64.6; 1.0% of total) were diagnosed with prostate cancer, of which two (12.5%; 95% CI 1.6-38.3; 0.3% of total) had clinically significant disease (ISUP Grade Group ≥2) (Table 1). In the immediate repeat-biopsy subgroup (n = 6; 1.0% of total), triggered by inadequate initial sampling (n = 3), PI-RADS 5 lesions (n = 2), or strong family history (n = 1), the cancer detection rate was 50.0% (3/6; 95% CI 11.8-88.2).

**Figure 3:**
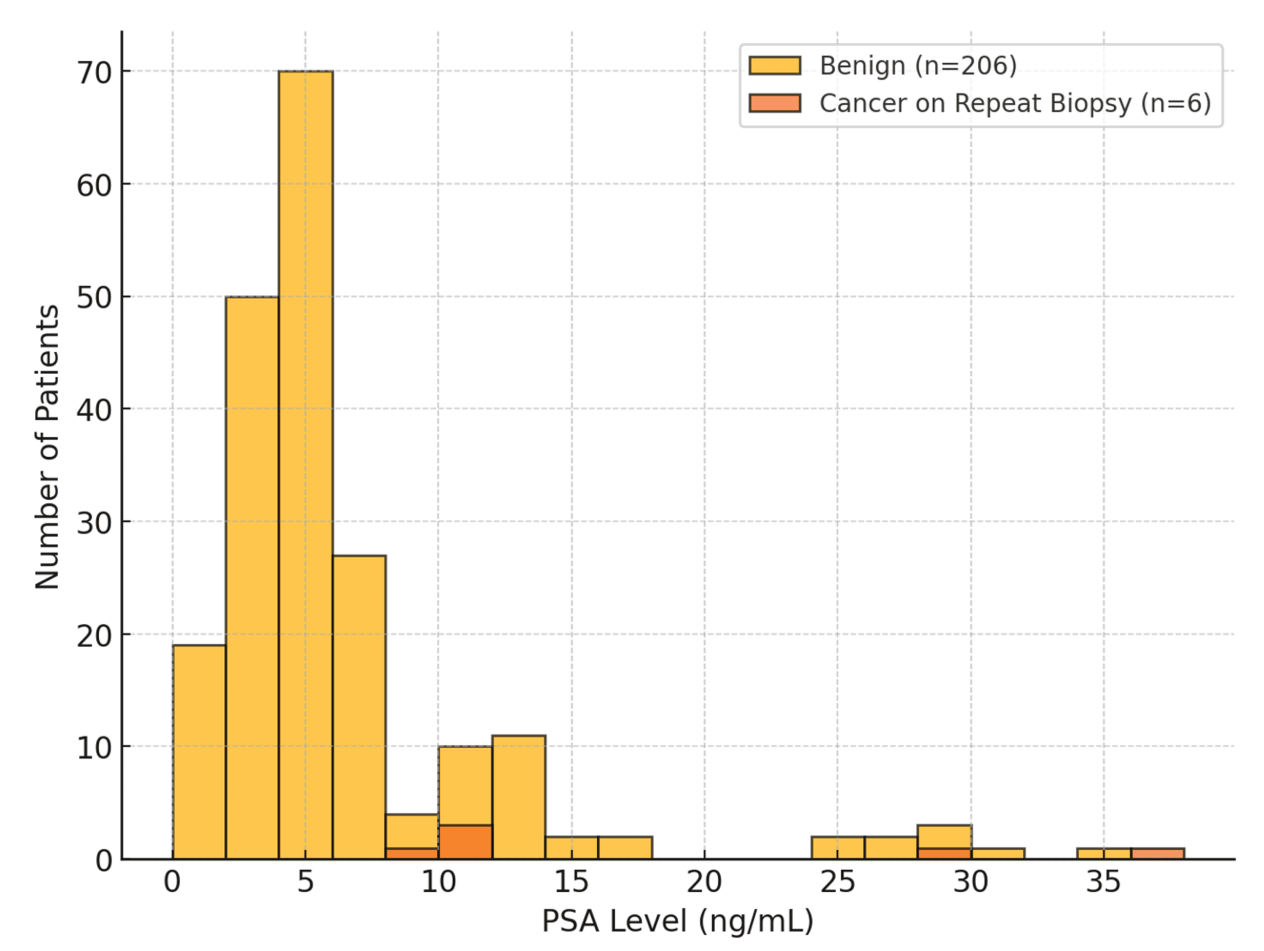
PSA distribution in patients with benign LATP results versus those diagnosed with cancer on repeat biopsy Histogram showing PSA levels in the benign cohort (n = 206) compared with patients later diagnosed with cancer on repeat biopsy (n = 6). All clinically significant cancers (ISUP Grade Group ≥2) occurred in men with PSA >10 ng/mL. ISUP, International Society of Urological Pathology; LATP, local anesthetic transperineal; PSA, prostate-specific antigen

PSA stratification showed that most men with benign LATP results (71.8%; 24.0% of total) had PSA 3-10 ng/mL, while 18.5% (6.0% of total) had PSA >10 ng/mL. All cancers detected on repeat biopsy occurred in men with PSA >3 ng/mL, and all clinically significant cancers were confined to those with PSA >10 ng/mL. Taken together (Table 2, Figure 3), PSA levels exceeding 10 ng/mL appeared to be a strong indicator for closer surveillance and timely repeat biopsy after an initial benign LATP result.

**Table 2:** PSA stratification in benign LATP biopsy patients and cancer detection on repeat biopsy Summary of PSA ranges among patients with benign LATP biopsy (n = 206) and their repeat-biopsy outcomes. Clinically significant cancers (ISUP Grade Group ≥2) were detected exclusively in patients with PSA >10 ng/mL. All clinically significant cancers on repeat biopsy occurred in patients with PSA >10 ng/mL. GG2, Gleason Grade Group 2; ISUP, International Society of Urological Pathology; LATP, local anesthetic transperineal; PSA, prostate-specific antigen

PSA range (ng/mL)	Number of patients (benign group)	% of benign group	Cancer diagnosed on repeat biopsy (n)	Clinically significant cancer (GG2+)
<3	20	9.70%	0	0
3-10	148	71.80%	1	0
10-20	28	13.60%	3	1
>20	10	4.90%	2	1

Prostatitis, ASAP, and HGPIN

Among patients with atypical or inflammatory findings, management and outcomes varied by histology. Of the 13 patients with ASAP (6.3% of the benign cohort, 2.1% of the full cohort), seven were monitored, five underwent repeat biopsy, and one was discharged. Repeat biopsy detected prostate cancer in two cases (40.0%; 95% CI 5.3-85.3), one of which was clinically significant (Grade Group 2). In the HGPIN subgroup (n = 8; 3.9% of the benign cohort, 1.3% of the full cohort), five patients were monitored, two underwent repeat biopsy, and one was discharged. One case of low-grade prostate cancer was identified (50.0%; 95% CI 1.3-98.7), but no clinically significant cancers were detected. By contrast, among the 37 cases of prostatitis (18.0% of the benign cohort, 6.0% of the full cohort), most (n = 31) were monitored with PSA checks; none required immediate repeat biopsy, and no cancers were subsequently detected during follow-up (0.0%; 95% CI 0.0-9.5).

Complications

LATP biopsy was well tolerated overall, with complications being rare across the 616 procedures. Pre-procedural antibiotics were administered in 12% of cases and post-procedural antibiotics in 13% as part of a targeted prophylaxis strategy. Four infectious events occurred (0.65%; 95% CI 0.18-1.66), comprising three cases of urosepsis and one case of epididymo-orchitis requiring readmission. All infectious events occurred in patients who had not received antibiotic prophylaxis; no infections were observed among those given either pre- or post-procedural antibiotics.

## Discussion

Benign findings following LATP prostate biopsy present an ongoing diagnostic challenge, largely due to the possibility of false-negative results. These may arise from tumor heterogeneity, inadequate sampling, or missed anterior lesions. As such, benign results should be interpreted alongside PSA kinetics, mpMRI findings, and other clinical indicators to guide further investigation.

The benign findings in LATP must also be considered in the context of false-negative rates. Historically, TRUS biopsies have been associated with substantial undersampling, particularly of the anterior and apical prostate. Landmark studies such as PROMIS highlighted that nearly half of clinically significant tumors were missed by TRUS systematic biopsy [2]. LATP mitigates this issue by enabling systematic and targeted sampling of the entire gland, including anterior zones, thereby reducing the likelihood of missing clinically significant disease. False-negative rates for LATP have been reported at <15-20% in several series, compared with up to 30-35% for TRUS [1,2]. The relatively low morbidity associated with LATP biopsy makes repeat biopsy a viable option when suspicion persists. In our study, 62.5% of repeat biopsies in patients with prior benign results remained benign, while 37.5% revealed prostate cancer, two of which (12.5%) were clinically significant (Gleason Grade Group ≥2). These findings highlight the importance of structured follow-up and targeted re-biopsy in high-risk individuals.

Notably, three of six patients (50%) who underwent immediate repeat biopsy were diagnosed with cancer. These biopsies were prompted by inadequate initial sampling, high PI-RADS scores, or strong family history. Among those diagnosed with cancer on repeat biopsy, 66.7% had a significant family history, and 33.3% had an abnormal DRE. Importantly, both clinically significant cancers were associated with PI-RADS 5 lesions. This emphasizes the utility of combining clinical, radiological, and familial risk factors to refine re-biopsy decisions.

Repeat biopsy was generally triggered by high-suspicion features, including PI-RADS 5 lesions or concerns about sampling adequacy. This introduces selection and verification biases that may inflate the cancer detection rate in the re-biopsy subgroup. These findings should therefore be interpreted with caution and not generalized to all patients with benign LATP results.

Our approach aligns with findings from Klemann et al., who showed that while most men with a benign initial biopsy do not develop cancer, a meaningful subset do, necessitating vigilant surveillance and individualized follow-up strategies [8]. They reported that approximately 14.2% of men with an initial benign TRUS biopsy were later diagnosed with prostate cancer, including 4.2% with clinically significant disease (Grade Group ≥2) during a median 10-year follow-up. Although their study involved TRUS rather than transperineal biopsy, which differs in sampling characteristics and false-negative rates, the findings underscore the potential for missed diagnoses and support structured, risk-adapted follow-up. Long-term follow-up studies, such as that by Palmstedt et al., have further shown that men with an initial benign biopsy generally have low prostate cancer mortality, reinforcing the value of risk-adapted surveillance strategies [9].

ASAP and HGPIN

ASAP represents a histological finding in which glandular proliferation demonstrates atypical features but is insufficient for a definitive cancer diagnosis [10]. It is clinically important due to its association with a higher likelihood of cancer on repeat biopsy. HGPIN, meanwhile, is widely considered a precursor to prostate cancer [11]. While not all patients with HGPIN progress, studies have shown a higher incidence of subsequent cancer in this group, particularly in those with elevated PSA or abnormal DRE. HGPIN often coexists with adjacent malignancy, underscoring the role of repeat biopsy in selected cases.

In our cohort, we identified 13 cases of ASAP and eight cases of HGPIN. These histological entities occupy a critical “grey zone” in prostate pathology, with distinct implications for cancer risk and follow-up. Our data confirm ASAP as a high-risk finding, with prostate cancer detected in 40% of re-biopsied patients, including one clinically significant tumor (Grade Group 2), echoing the 45.1% rate reported by Kim et al. [12]. This supports guideline recommendations for early re-biopsy (typically within three to six months). By contrast, HGPIN carried a lower cancer yield, with only one case of low-grade (Grade Group 1) prostate cancer detected on repeat biopsy. While older TRUS-based studies prompted early re-biopsy in all cases of HGPIN, contemporary practice increasingly favors PSA and MRI surveillance, reserving re-biopsy for men with PI-RADS 4-5 lesions or rising PSA. Our findings support this MRI-informed approach, which spares low-risk men unnecessary procedures [11,13].

Comparison with landmark TRUS data

Our findings can be interpreted in the context of long-term outcomes reported in landmark TRUS biopsy cohorts, such as the study by Klemann et al. [8], which followed over 37,000 men for two decades after an initial benign TRUS biopsy. That study demonstrated that a benign biopsy carries important prognostic information, with prostate cancer-specific mortality remaining extremely low, particularly among men with PSA ≤10 ng/mL. Similar findings have been reported by Beckmann et al. and Lewicki et al. [14,15].

Lewicki et al. examined more than 7,000 men enrolled in a large screening trial and showed that a negative biopsy substantially reduced the future risk of clinically significant cancer and prostate cancer-related mortality [15]. However, their data also highlighted that risk was not eliminated, particularly in men with persistently elevated PSA or adverse baseline features, underscoring the importance of continued vigilance.

Beckmann et al. further extended this concept to the active surveillance population, demonstrating that men with negative confirmatory biopsies had markedly lower rates of disease progression compared with those with positive biopsies [14].

While our LATP cohort has shorter follow-up, the principle is similar: a benign biopsy generally indicates low future risk, especially in men with stable PSA. However, TRUS studies also underscore the false-negative burden; cancers missed on the initial biopsy accounted for later diagnoses, with repeat TRUS biopsies detecting cancer in 10-34% of cases. LATP addresses many of TRUS’s limitations, offering improved anterior and apical sampling and incorporating MRI-targeted cores [1,16].

The diagnostic advantages of LATP over TRUS are well documented. The systematic review by Kanagarajah et al. reported favorable cancer detection rates and significantly lower infection risks with LATP compared to the transrectal approach [16]. Building on this, the multicenter TRANSLATE trial by Bryant et al. demonstrated a statistically significant increase in the detection of Grade Group ≥2 disease using LATP compared with TRUS in biopsy-naïve men (60% vs 54%; p = 0.031), along with a reduced risk of post-procedure infection requiring admission [1]. These findings strengthen the case for LATP as a first-line diagnostic strategy and have supported its adoption in updated clinical guidelines.

Long-term data from the Prostate Testing for Cancer and Treatment (ProtecT) trial further support a conservative, risk-stratified follow-up approach, showing that prostate cancer-specific mortality remained low (~2-3%) at 15 years regardless of initial treatment, despite higher rates of disease progression and metastases in the monitoring group [17]. In the context of benign LATP results, these findings reinforce the safety of surveillance in appropriately selected men, while emphasizing the importance of vigilant follow-up in those at higher risk of harboring undetected clinically significant cancer. Nonetheless, the substantial proportion of benign LATP biopsies highlights the ongoing need to refine follow-up strategies, an area still lacking standardized guidance in clinical practice.

Our data, showing cancer detection in 37.5% of repeat biopsies after a benign LATP, suggest that even with a more accurate technique, missed cancers remain clinically relevant, though likely fewer and more easily identified with structured PSA- and MRI-driven surveillance.

Beyond established imaging and PSA-based monitoring, emerging biomarkers such as those incorporated into the Michigan Prostate Score (MPS) show promise for refining post-biopsy surveillance. MPS integrates urinary PCA3 and TMPRSS2:ERG expression with serum PSA to improve specificity for clinically significant prostate cancer [18]. Incorporating such biomarkers into follow-up pathways after benign LATP results may help identify patients who truly warrant re-biopsy, thereby reducing patient burden and healthcare costs.

Limitations

This study has several limitations. First, it was conducted at a single center, which may limit generalizability. Second, the sample size, particularly for patients undergoing repeat biopsy, was relatively small, reducing statistical power. Third, the follow-up period was short, and many patients in the monitored cohort remain under active surveillance. This limits our ability to assess the negative predictive value of LATP and prevents a full comparison of long-term outcomes between monitored and re-biopsied groups. In addition, repeat biopsy was more likely in patients with high PI-RADS scores or other concerning features, introducing selection and verification bias that may have inflated the cancer detection rate in these enriched subgroups. We were also unable to robustly assess PSA velocity due to the limited monitoring interval. Finally, the study was descriptive in design and not adjusted for potential confounders; therefore, the findings should be regarded as exploratory and hypothesis-generating, serving as a basis for future prospective research rather than definitive conclusions. Multicenter prospective studies with standardized follow-up protocols and longer observation periods are needed to validate these findings and refine follow-up strategies after benign LATP biopsy.

## Conclusions

LATP prostate biopsy is a safe, effective, and well-tolerated diagnostic approach that improves anterior gland sampling and significantly reduces infection risk compared with the traditional transrectal route. However, a benign biopsy does not definitively exclude malignancy, and the relatively short follow-up in our study limits firm conclusions about the negative predictive value of LATP. This highlights the need for structured, risk-adapted surveillance. Our single-center experience suggests that integrating PSA kinetics, mpMRI, and key clinical risk factors can help guide decisions on repeat biopsy, in line with contemporary international guideline recommendations.

Future research should focus on refining follow-up protocols and clarifying the role of advanced imaging and emerging biomarkers, such as the MPS, to reduce unnecessary re-biopsies. From a health system perspective, incorporating these tools within standardized discharge criteria for low-risk men after a benign LATP result could help reduce follow-up burden in secondary care while concentrating resources on those at highest risk. With longer-term validation, a streamlined MRI-guided pathway has the potential to enhance cancer detection efficiency, minimize false negatives, improve patient reassurance, and optimize diagnostic service capacity.
